# Liver Fibrosis in Non-alcoholic Fatty Liver Disease: From Liver Biopsy to Non-invasive Biomarkers in Diagnosis and Treatment

**DOI:** 10.3389/fmed.2021.615978

**Published:** 2021-04-14

**Authors:** Leen J. M. Heyens, Dana Busschots, Ger H. Koek, Geert Robaeys, Sven Francque

**Affiliations:** ^1^Faculty of Health and Life Sciences, Hasselt University, Hasselt, Belgium; ^2^School of Nutrition and Translational Research in Metabolism, NUTRIM, Maastricht University, Maastricht, Netherlands; ^3^Department of Gastro-Enterology and Hepatology, Ziekenhuis Oost-Limburg, Genk, Belgium; ^4^Division of Gastroenterology and Hepatology, Department of Internal Medicine, Maastricht University Medical Centre, Maastricht, Netherlands; ^5^Department of Gastroenterology and Hepatology, University Hospital Katholieke Universiteit (KU) Leuven, Leuven, Belgium; ^6^Department of Gastroenterology and Hepatology, Antwerp University Hospital, Antwerp, Belgium; ^7^Laboratory of Experimental Medicine and Paediatrics, Faculty of Medicine and Health Sciences, University of Antwerp, Antwerp, Belgium

**Keywords:** NAFLD, liver fibrosis, liver biopsy, non-invasive assessment, liver stiffness

## Abstract

An increasing percentage of people have or are at risk to develop non-alcoholic fatty liver disease (NAFLD) worldwide. NAFLD comprises different stadia going from isolated steatosis to non-alcoholic steatohepatitis (NASH). NASH is a chronic state of liver inflammation that leads to the transformation of hepatic stellate cells to myofibroblasts. These cells produce extra-cellular matrix that results in liver fibrosis. In a normal situation, fibrogenesis is a wound healing process that preserves tissue integrity. However, sustained and progressive fibrosis can become pathogenic. This process takes many years and is often asymptomatic. Therefore, patients usually present themselves with end-stage liver disease e.g., liver cirrhosis, decompensated liver disease or even hepatocellular carcinoma. Fibrosis has also been identified as the most important predictor of prognosis in patients with NAFLD. Currently, only a minority of patients with liver fibrosis are identified to be at risk and hence referred for treatment. This is not only because the disease is largely asymptomatic, but also due to the fact that currently liver biopsy is still the golden standard for accurate detection of liver fibrosis. However, performing a liver biopsy harbors some risks and requires resources and expertise, hence is not applicable in every clinical setting and is unsuitable for screening. Consequently, different non-invasive diagnostic tools, mainly based on analysis of blood or other specimens or based on imaging have been developed or are in development. In this review, we will first give an overview of the pathogenic mechanisms of the evolution from isolated steatosis to fibrosis. This serves as the basis for the subsequent discussion of the current and future diagnostic biomarkers and anti-fibrotic drugs.

## Introduction

Non-alcoholic fatty liver disease (NAFLD) refers to the presence of liver steatosis in the absence of factors that are known to induce lipid accumulation in hepatocytes, such as alcohol consumption or the use of steatogenic drugs. The diagnosis requires > 5% of the hepatocytes containing lipid droplets on histology or the amount of fat in the liver to exceed 5% of the total liver weight (1). Currently, NAFLD is the most common chronic liver disease with an estimated prevalence of 25% worldwide (2). The prevalence of NAFLD parallels the global increase in overweight and obesity which is the result of an increase of caloric intake over expenditure that leads to an increase in body mass index (BMI) (3). As a result, NAFLD will become the most common cause of liver cirrhosis, decompensated liver disease or hepatocellular carcinoma (HCC) in the short term, and thus the most important indication for liver transplantation (4).

**Figure 1:**
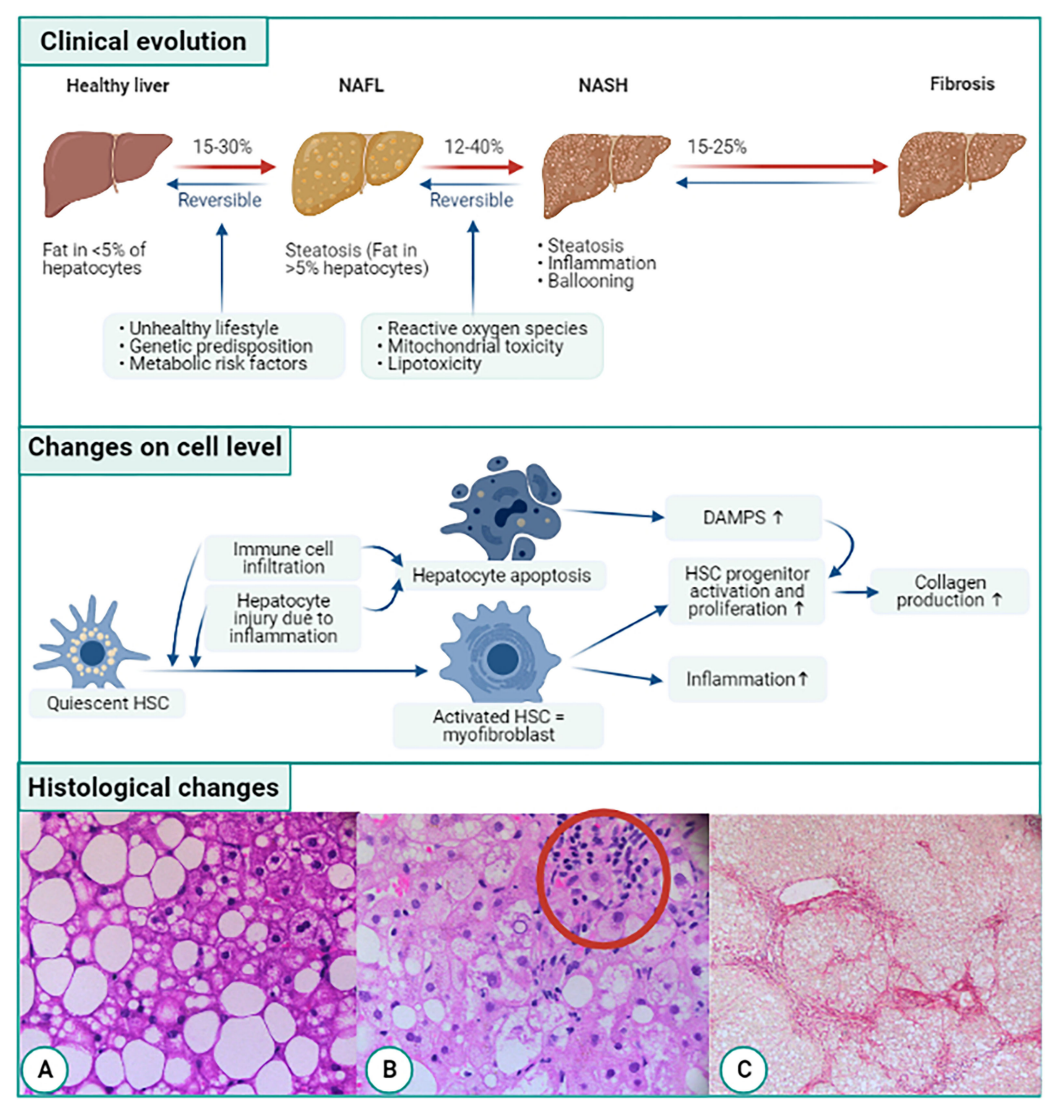
Overview of evolution of NAFLD related fibrogenesis on clinical, cellular, and histological level. On the clinical level NAFLD starts of as simple steatosis (NAFL). The abnormal amount of liver fat triggers inflammation by infiltrating immune cells and secretion of cytokines. This is called non-alcoholic steatohepatitis or NASH which can cause liver fibrosis. On cellular level, quiescent hepatic stellate cells (HSCs) are activated by immune cell infiltration and hepatocyte injury due to inflammation. The activated HSC transdifferentiates into collagen producing myofibroblasts furthermore the myofibroblasts trigger HSC progenitor proliferation and activation. Another consequence of the immune cell infiltration and hepatocyte injury is apoptosis of hepatocytes, leading to the release of damage-associated patterns (DAMPs). DAMPs also activate hepatic progenitor cells. Both the myofibroblasts and HSCs will start producing collagen. On a histological level, first fat accumulates in the liver (A). This leads to the infiltration of immune cells (B) and ballooning and eventually liver fibrosis occurs (C). Histological pictures courtesy of Dr. P. Van Eyken, pathologist, Ziekenhuis Oost-Limburg, Genk, Belgium.

NAFLD comprises a broad spectrum of liver lesions but also has extra-hepatic consequences. These extra-hepatic complications, including cardiovascular disease, diabetes, and non-liver malignancies, are responsible for a significant part of NAFLD-attributable morbidity and mortality (5, 6). Furthermore, there is a considerable impact on the quality of life (7, 8). With respect to the liver, the NAFLD spectrum consists of the following entities: isolated fatty liver (non-alcoholic fatty liver, NAFL); and non-alcoholic steatohepatitis (NASH), i.e., steatosis accompanied by chronic inflammation and cell damage, histologically characterized by lobular inflammation and ballooning of hepatocytes, the latter being the driving force of fibrosis that can evolve to cirrhosis and decompensated cirrhosis (Figure 1). HCC can also develop, even in non-cirrhotic NAFLD (9). The rate of disease progression is usually slow. About 20% of patients with NAFLD will develop NASH in three to seven years (10), which is considered the potentially progressive form of the disease (11). About 9 to 25% of individuals with NASH develop cirrhosis over a 10 to 20 year period (12).

Unfortunately, it is very hard to know which NASH patient will progress to cirrhosis due to the complex multifactorial etiology of NASH determined by genetic, epigenetic, lifestyle, and nutritional factors (13). However, the stage of liver fibrosis is the strongest predictor for liver-related mortality and development of other comorbidities (14–18), with an increase if fibrosis (F) is ≥ 2 on a scale of 0–4 as proposed by the NASH Clinical Research Network Scoring System (NASH CRN) (19). Accordingly, NASH patients with ≥F2 are considered the target population for pharmacological treatment and are most likely to benefit from antifibrotic drugs. Regression of stage F1 fibrosis is more likely with simple lifestyle changes and treatment of the metabolic comorbidities (20). Therefore, it is important to correctly diagnose the stage of liver fibrosis, preferably with non-invasive methods. Additionally, regression of advanced fibrosis should be the primary hepatic endpoint in clinical studies for antifibrotic drugs (21). Therefore, this review will tackle diagnostic methods to determine the stage of liver fibrosis and antifibrotic drugs that can reduce advanced fibrosis.

## Pathogenesis

As understanding the pathogenesis of NAFLD and NAFLD-related fibrosis is vital in the development of biomarkers for its diagnosis and in finding targets for its treatment, we first review the most important aspects of NAFLD-related fibrogenesis.

### From Liver Steatosis to NASH

Weight gain, often caused by an unhealthy lifestyle with a high-calorie diet and decreased physical activity, is one of the most important factors in the development of NAFLD. The liver plays a critical role in maintaining the metabolic balance that comes under pressure with a high caloric intake and low energy expenditure. Lipid overload, as seen in NAFLD, is a major contributor to the development of lipotoxicity (Figure 1A). Lipotoxicity accelerates the development of progressive inflammation, oxidative stress, and fibrosis (22). The excess energy consumed is usually stored in the form of fat in both subcutaneous and visceral depots. This capacity of the adipose tissue to store fat is genetically determined. When this capacity is exceeded, the adipose tissue experiences an overload and becomes damaged. This results in adipose tissue insulin resistance (IR) and inflammation of the tissue with imbalances in the secretion of adipokines and other inflammatory mediators (23), which causes a low-grade systemic inflammation (24). Together with ectopic fat accumulation, this leads to IR in the muscles and liver (25). The IR causes a disbalance in the homeostasis of glucose and lipid metabolism. As a result, more free fatty acids (FFA) that have to be processed by the liver, enter the circulation (26). Another consequence is that dietary carbohydrates (especially fructose) are absorbed by the liver and converted to FFA by *de novo lipogenesis*. About 40% of the fat that builds up in the liver comes from dietary carbohydrates and fat. The other 60% is derived from the dysfunctional adipose tissue (26). Thus, IR leads to an increase in FFA flux, leading to a toxic effect on the liver (27). The FFA are normally broken down in the mitochondria by beta-oxidation. Due to the FFA overload, the mitochondria are overwhelmed, and this leads to mitochondrial uncoupling. As a result, they produce reactive oxygen species (ROS) (22, 26). Combined with the dysfunctional adipose tissue and endotoxins from the gut, this leads to a pro-inflammatory and apoptotic climate in the liver, causing NASH (26–28). The Kupffer cells, the resident macrophages of the liver, as well as infiltrating immune cells, contribute to the inflammatory state of NASH. Kupffer cells absorb large amounts of FFA, which drives them toward an inflammatory phenotype. This leads to the secretion of inflammatory cytokines such as interleukin (IL)-6, tumor necrosis factor (TNF)-α and IL-10. Both IL-6 and TNF-α are associated with NASH progression (29, 30).

Taken together, NASH is the result of a complex interplay between different factors like genetic variation and obesity, which leads to a profibrotic climate in the liver (31).

### From NASH to Liver Fibrosis

Immune responses in chronic liver diseases like NAFLD, not only lead to the restoration of tissue function but also to tissue injury. An overactive or exaggerated immune response can result in organ dysfunction and leads to the deposition of fibrotic tissue in parallel to the cell loss (31). These immune responses comprise both innate and adaptive responses (32). For example, neutrophil infiltration is often seen in histologic samples of NASH patients (Figure 1B). Additionally, patients with NASH and advanced fibrosis related to NASH have a higher neutrophil/lymphocyte ratio than patients without NASH (33, 34). Likewise, CD8+ lymphocytes have also been seen in the inflammatory infiltrate in NASH (35).

The inflammation caused by NASH causes hepatocyte death and apoptosis. The dying hepatocytes release damage-associated molecular patterns (DAMPs). The DAMPs, including nucleic acids, intracellular proteins, and adenosine triphosphate (ATP), send a danger signal to the surrounding cells (36). The danger signal activates the hepatic progenitor cells (HPCs). Apoptosis, on the other hand, produces low levels of DAMPs since most of the cell content will be retained in an apoptotic body. These apoptotic bodies will be phagocytosed by hepatic stellate cells (HSCs) and Kupffer cells. This induces a pro-fibrogenic response. Additionally, the DNA from the apoptotic hepatocytes triggers the activation of Toll-Like-Receptor (TLR)-9 on HSCs and collagen production (37).

Infiltration of the immune cells activates the trans-differentiation of HSCs into collagen-producing myofibroblasts (28). Usually, this process is involved in tissue repair upon short-term injury. When liver injury occurs, the HSCs are activated and differentiate from the quiescent phenotype to proliferative and contractile myofibroblasts (38). In their quiescent stage, HSCs store retinoids and synthesize glial fibrillary acidic protein (GFAP). When activated, a gradual loss of retinoids and GFAP coincides with their development into myofibroblasts with the synthesis of extracellular matrix (ECM) products like type I, type III, and type IV collagen but also hyaluronic acid (HA) (39). Levels of the glycosaminoglycan polymer HA increase with the amount of liver fibrosis (40). The collagen accumulation is accompanied by a rise in metalloproteinases (MMPs) such as MMP-9, which break down ECM products (41, 42). The combination of active and overexpressed MMP-9 and build-up of type III collagen leads to an abundance of cleaved type III collagen products like plasma N-terminal propeptide of type III procollagen (PIIIPN) or neo-epitope PRO-C3 (43, 44). Normally the MMPs are kept in check by tissue inhibitors of metalloproteinase (TIMPs). There are four TIMPs of which TIMP-1 is secreted by macrophages and fibroblasts (45). In murine fibrotic livers, likewise to the increase in MMPs, high concentrations of TIMP-1 were found (46). This creates a disturbance in the MMP/TIMP balance and, therefore a shift toward ECM synthesis and thus fibrogenesis (47). Alpha-2 macroglobulin (A2M) causes the balance to tip even further toward fibrogenesis by also inhibiting the MMPs (48). In addition to the ECM products, myofibroblasts also synthesize α-smooth muscle actin (α-SMA) (49). Ramzy et al. indicated that an increase in α-SMA marks the activation of HSCs (50).

During differentiation, the characteristic star-like shape of the HSCs changes to a more droplet form. The process is then balanced by the counteracting anti-fibrotic mechanisms resulting in the inactivation or apoptosis of the myofibroblasts and scar resolution. In chronic diseases, like NAFLD, there is an imbalance in these processes. The imbalance will cause persistent activation of proliferating, contractile, and migrating fibroblasts. This leads to the excessive production of ECM. The abundance of ECM will destroy the physiological architecture of the liver (51). The regulators of this balance are non-parenchymal cells (NPCs) like Kupffer cells and other immune cells, which are, as mentioned above, recruited to the site by the death and apoptosis of hepatocytes (52). NPCs will start producing pro-fibrogenic cytokines. On a molecular basis, a complex network of cytokine-induced pathways arises to coordinate the pro-fibrogenic cell interactions. The proposed signaling pathways associated with HSC activation and fibrosis progression are the transforming growth factor beta (TGF-β), platelet-derived growth factor (PDGF), inflammasome (NLRP3)-caspase 1, and the WNT/β-catenin (28).

### From Liver Fibrosis to Liver Cirrhosis

Progression of liver fibrosis to liver cirrhosis varies between people depending on environmental and host factors (53). Cirrhosis is a consequence of long-standing fibrogenesis that results in the encapsulation of injured liver parenchyma by a collagenous scar. Histologically, cirrhosis is characterized by fibrotic septa that connect the portal tracts with each other and with the central veins (Figure 1C). This leads to a disconnection of the hepatocytes from the central vein, creating islands of hepatocytes. Vascular changes also occur, including loss of sinusoidal fenestrae and appearance of a basal membrane, or so-called capillarisation of the liver sinusoids, another hallmark of cirrhosis (54). The changes in liver structure ultimately lead to an increase of intravascular resistance within the portal system and decreased hepatic perfusion (55). The consequence is a loss of liver function (56).

### Molecular Signaling Pathways Involved in Liver Fibrogenesis

#### TGF-β Signaling

TGF-β, together with PDGF, is the most potent inducer of hepatic fibrosis (57). The TGF-β superfamily consists of 33 members, of which TGF-β1 plays an essential role in liver fibrogenesis (58). The consequences of TGF-β1 signaling are inhibition of HSC apoptosis and induction of HSCs to produce excessive amounts of ECM proteins like fibronectin and collagen types I, II, and IV (59). Additionally, the production of matrix-degrading proteins is inhibited by TGF-β1 (60). In patients with hepatic fibrosis, increased concentrations of TGF-β1 correlate with the severity of fibrosis (61, 62).

TGF-β1 mainly exerts its effects via small Mothers Against Decapentaplegic (SMAD)-dependent pathways. The SMAD family is divided into three groups based on their functions. First, there are the receptor-regulated SMADs (R-SMADs), which include SMAD1, SMAD2, SMAD3, SMAD5, and SMAD8. Secondly, SMAD4 is the only member of the common SMAD (co-SMAD). The third group consists of the inhibitory SMADs (i-SMADs) and includes SMAD6 and SMAD7. The R-SMADs bind to membrane bound serine/threonine receptors and are activated by their kinase activity. Co-SMADs act as co-factors and attach to the R-SMADs to form a complex that will translocate to the nucleus of the cell. i-SMADs, on the other hand, counteract the effect of the R-SMADs (63). SMAD3 and SMAD4 have been found to be pro-fibrotic, whereas SMAD2 and SMAD7 are protective (64). SMAD3 induces hepatocyte death and lipid accumulation, especially in NASH (65). SMAD4 even enhances fibrogenesis by promoting SMAD3 activity. SMAD7, on the other hand, downregulates SMAD3 (66, 67). On their turn, SMADs also act as signal integrators and interact with the mitogen-activated protein kinases (MAPK) and nuclear factor kappa beta (NFκB) pathway (68).

#### PDGF Signaling

PDGF is a growth factor that promotes HSCs division and proliferation (28). The PDGF family consists of four members: PDGF-A, -B, -C, and -D (69). In healthy circumstances, PDGF is produced by platelets. When liver injury occurs, Kupffer cells recruited to the site of inflammation secrete PDGF (70). All PDGF members and its receptors (PDGF-R) are overexpressed in the case of liver fibrosis, and the activity increases with the degree of liver fibrosis (71–74). For example, PDGF-C activates the TGF-β/SMAD3 pathway in mice, leading to HSC proliferation, collagen production, and eventually fibrosis (72). However, of the four members, PDGF-B and -D are the most potent in activating the downstream pathways extracellular signal-regulated protein kinase/mitogen-activated protein kinase (Erk/MAPK) and protein kinase B (Akt/PKB). The activation leads to HSC proliferation (71, 75). PDGF-A expression was increased in hepatocytes from fibrotic livers compared to normal livers (76). In HSCs, on the other hand, although they express both receptors, only PDGF-BR expression was upregulated during HSC activation both *in vitro* as *in vivo* (77, 78).

#### The NLRP3 Inflammasome Caspase-1 Pathway

Inflammasomes are multiprotein complexes that sense danger signals like DAMPs and pathogen-associated molecular patterns (PAMPs) from damaged cells and pathogens (79). There are multiple inflammasomes implicated in liver disorders, but the nucleotide-binding oligomerization domain (NOD)-like receptor protein 3 (NLRP3) inflammasome has been studied most extensively (80–82). The NLRP3 inflammasome is activated in a two-step process. First, a bacterial signal, for example lipopolysaccharide (LPS), upregulates Nlrp3 expression via the NFκB-pathway (83). This, in turn, will enable a second signal, e.g., a DAMP, to activate the NLRP3 inflammasome. Once activated, the inflammasome binds with the adaptor molecule ASC to mediate caspase-1 cleavage, thereby activating the enzyme (79, 84). Caspase-1 activates pro-inflammatory cytokines IL-1β and IL-18 by proteolysis, though also activates the cytosolic protein gasdermin D (GSDMD) (85). GSDMD in a cleaved form will create pores in the plasma membrane of cells (86). This induces pyroptotic cell death and, consequently, the release of IL-1β and IL-18 (86–88).

In NAFLD, the NLRP3 inflammasome has been found to negatively regulate disease progression (89). In early NAFLD models, mRNA upregulation of the NLRP3 inflammasome components, like *Nlrp3, Asc*, and *Casp1*, was found. However, no active inflammasomes were found, indicating that not enough signals were present in a fatty liver to properly activate the inflammasome (90, 91). In NASH, on the other hand, IL-1 β will stimulate the production of inflammatory cytokines, thus aggravating the already existing inflammation (92). In a mouse knock-in model of the NLRP3 inflammasome, inflammation was increased, and simultaneously a high neutrophil infiltration was found. In addition, NLRP3 also induced HSC activation and collagen deposition, thereby causing liver fibrosis (80). Blockage of NLRP3 resulted in a reduction of liver inflammation and fibrosis in an experimental mouse model of NASH (93). It is clear that NLRP3 is involved in the pathogenesis of liver fibrosis with NAFLD. Nevertheless, additional studies are necessary to provide a better insight into these mechanisms.

#### Wnt/β-Catenin Signaling

The Wnt signaling pathway consists of canonical and non-canonical arms and regulates a large number of cellular functions (94). The canonical pathway exerts anti-lipid formation and anti-inflammatory effects, while the non-canonical pathway promotes fat formation, lipid accumulation, and inflammation (95). An imbalance between these two pathways has been associated with NAFLD by triggering lipotoxicity and fibrogenesis (96, 97). More specifically, the Wnt signaling pathway promotes hepatic fibrosis by enhancing HSC activation and survival, and upregulation of TGF-β/SMAD pathways (49).

### Other Mechanisms That Contribute to Liver Fibrogenesis

#### Gut Liver Axis

About 70% of the liver's blood supply comes from the intestines. This blood circulation enables the liver to interact with products derived from the intestines, like bacterial DNA, LPS, or intact bacteria due to an increased intestinal barrier permeability (98). Normally, the Kupffer cells will clear the endotoxins, maintaining the immune tolerance and homeostasis. Alteration of the gut microbiome, gut permeability, and Kupffer cell responsivity can alter this balance (31). Moreover, fructose, a compound frequently found in sugar beverages, has been shown to promote a leaky gut and liver fibrosis. Fructose induces the ethanol-inducible cytochrome P450-2E1-mediated oxidative and nitrative stress (99). In addition, the bacterial products can bind to the TLRs in the liver, thereby inducing liver inflammation. This causes the progression of liver disease due to the fact that the TLRs will activate the NFκβ and the c-Jun N-terminal kinase (JNK) pathways (100). Most of the studies are, however, performed in mice, and more research in humans is necessary (101). A study conducted by Kapil et al. in humans indicated that small intestinal bacterial overgrowth and TLR signaling are involved with liver histology in NAFLD (102). In a study by Boursier et al. it was shown that NAFLD severity was associated with gut microbiome alterations and shifts in the metabolic function of the microbiome (103). More specifically, they found that the *Ruminococcus* bacteria were independently associated with fibrosis (103). These first results in human trials concerning the microbial environment are leading to further investigations of the influence of the gut microbiome in people with metabolic disorders like NAFLD (104).

#### Genetic Mechanisms

In addition to environmental factors, genes play a role in NAFLD (1). Several genes have been identified through genome-wide association studies (105–107). Amongst those, Patatin-like phospholipase domain containing 3 (PNPLA3) and transmembrane 6 superfamily member 2 (TM6SF2) seem to have the biggest impact (108). The PNPLA3 gene has been most extensively studied. It is located on chromosome 22 and encodes a 481 amino acid protein that mediates triacylglycerol hydrolysis. The I148M variant of PNPLA3 (rs738409) is strongly associated with NAFLD in adults but also in obese children and adolescents (109, 110). In a mouse model of NAFLD, overexpression of the I148M variant of the PNPLA3 gene caused hepatic steatosis (111). However, the exact mechanism is not yet known (105). The TM6SF2 gene, located on chromosome 19, plays a role in the progression of NAFLD. A single nucleotide polymorphism (rs58542926) replacing a cytosine by a thymine in position 167 has been linked to an increased hepatic triglyceride content (112). This specific gene variant has also been associated with fibrosis progression (113). Both PNLPA3 and TM6SF2 thus exert an additive effect on NASH and significant fibrosis (114).

## Diagnosis of Liver Fibrosis

As previously stated, the stage of fibrosis is the most essential determinant of liver-related progression and mortality, and a key indicator for the development of other comorbidities like type 2 diabetes (T2DM) and cardiovascular disease, indicating the need to correctly diagnose fibrosis (115).

### Liver Biopsy

Liver biopsy is currently considered as the gold standard for the diagnosis and histological assessment of NAFLD (1). Unfortunately, due to its invasive nature, a biopsy is not suited for screening purposes and cannot be implemented early in the diagnostic path of potential patients (116). It is mostly reserved for patients with a high risk of advanced liver disease during long-term follow-up, to distinguish NASH from NAFL and to determine the extent of liver fibrosis (117, 118). Additionally, a biopsy is still required in more advanced stages of drug development for NASH to assess treatment efficacy (119, 120).

#### Histological Scoring Systems for Liver Biopsy Samples

There are different histological scoring systems for classifying liver biopsy samples (121). However, the most widely used scores are the NASH CRN including the NAFLD Activity Score (NAS) and the Steatosis-Activity-Fibrosis (SAF) score (Table 1) (19, 122). The NAS scoring system is initially developed for use in clinical studies, and a definition of NASH has been based on this score. The score ranges from 0 to 8 (Table 1) and is composed of the unweighted sum of steatosis, ballooning, and lobular inflammation. A score between 0 and 2 corresponds to no NASH, 3–4 is borderline NASH, and definite NASH has a score between 5 and 8. Yet, there are several remarks concerning this scoring system. Firstly, this definition of activity does not distinguish steatosis separately from necroinflammation. Secondly, lobular inflammation outweighs ballooning, while ballooning is an essential feature of the NASH definition. Thirdly, the grading of the ballooning is based on the number of ballooned cells, without a clear definition of how to assess ballooning. This causes a greater opportunity for interobserver variability in NASH diagnosis. The SAF score, on the other hand, assesses steatosis (S) separately from activity (A), and of course, also fibrosis (F). This scoring system was developed by the Fatty Liver: Inhibition of Progression (FLIP) consortium. The activity score is, in this case, a combination of lobular inflammation and ballooning both scored from 0 to 2, overcoming the problem of one criterium outweighing the other. Additionally, a clear definition of ballooning is given. If the size of the hepatocyte is twice as big as usual, it is considered as severe ballooning (123). Although both the NAS and SAF score have a comparable fibrosis grading system, the SAF score may potentially be more appropriate for routine diagnosis and clinical trials as it comes with an easy to use diagnostic algorithm and better-defined criteria leading to less interobserver variability (117). However, future comparative studies are needed to determine which scoring system is the most potent in scoring NAFLD related fibrosis. In contrast to differences in the concepts of activity and the scoring of the features of ballooning and lobular inflammation, the scoring of fibrosis is the same in both NASH CRN and FLIP SAF (except for the subclassification of F1 in NASH CRN). Stage one (F1) of NASH CRN system is composed of three subclasses, namely: F1a stands for mild perisinusoidal/pericellular fibrosis, F2a is moderate perisinusoidal/pericellular, and F1c is portal/periportal fibrosis. For the FLIP SAF system, the subclasses of F1 were pooled into one stage of mild perisinusoidal/pericellular fibrosis. Stage two (F2) correlates with perisinusoidal/pericellular and portal/periportal fibrosis. Next, stage three (F3) corresponds to bridging fibrosis. Lastly, stage four (F4) stands for liver cirrhosis. In a clinical situation, people speak of significant and advanced fibrosis; in this case significant fibrosis stands for ≥F2 and advanced for ≥F3. It is thus different from the MetaVir score designed for the staging of liver fibrosis caused by viral hepatitis where F1 does not have subclassifications, and for the other stage's fibrosis expansion should be located in the portal zones (124, 125). Consequently, when reviewing literature, one should pay attention to the scoring system used as a reference golden standard when studying non-invasive biomarkers.

**Table 1:** Comparison between the histologic scoring of NAFLD according to NASH CRN system and SAF system (18, 120).

	**Score**	**NASH-CRN**	**SAF**	**Score**
Steatosis	0	<5%	<5%	0
	1	5–33%	5–33%	1
	2	>33–67%	>33–67%	2
	3	>67%	>67%	3
Lobular inflammation	0	No foci	No foci	0
	1	<2 foci/20X	<2 foci/20X	1
	2	2–4 foci/20X	>2 foci/20X	2
	3	>4 foci/20X		
Ballooning	0	No ballooning	Normal hepatocytes	0
	1	Few ballooned cells	Clusters of rounded, pale hepatocytes	1
	2	Many ballooned cells	Many enlarged (2X normal size) hepatocytes	2
Fibrosis	0	No fibrosis	No fibrosis	0
	1	1a Mild, zone 3 perisinusoidal/pericellular fibrosis 1b Moderate, zone 3 perisinusoidal/pericellular fibrosis 1c Portal/periportal fibrosis	Mild fibrosis perisinusoidal/pericellular	1
	2	Perisinusoidal/pericellular and portal/periportal fibrosis	Perisinusoidal/pericellular and portal/periportal fibrosis	2
	3	Bridging fibrosis	Bridging fibrosis	3
	4	Cirrhosis	Cirrhosis	4
Composite score for activity	0–8	NAS = NAFLD Activity Score = steatosis + ballooning + lobular inflammation	A = ballooning + lobular inflammation	0-4

*NAS, NAFLD Activity Score; NASH CRN, Non-Alcoholic steatohepatitis clinical research network scoring system; SAF, Steatosis-Activity-Fibrosis*.

Outside these commonly used scoring systems, there are also other more granular scoring systems though they are not used as the golden reference standard in studies with non-invasive biomarkers. For instance, the Ishak staging system ranging from 0 to 6 with 6 being cirrhosis, was previously one of the most frequently used fibrosis scoring systems in clinical trials for different etiologies of liver disease. The Ishak fibrosis stages reflect more scarring than each preceding stage. Succession from one stage to the next represents progressively more advanced liver disease (126). Another scoring system is the EPoS staging system developed by the Elucidating (E) Pathways (P) of (o) Steatohepatitis (S) consortium. This system is based on e-slides, histological glass slide images that have been turned into electronic files. It includes, similarly to the Ishak system, seven stages ranging from 0 to 6. In a first study presented at the International Liver Congress of 2018, the EPoS scoring system showed promising results in terms of interobserver reproducibility (127).

#### Limitations of a Liver Biopsy

Although being the golden standard, a liver biopsy also has several limitations. The procedure comes with some discomfort and risks. As for the incidence of pain, this was reported to be 20%, though when a mildly unpleasant feeling was included in the assessment, the incidence increased to 84% (128). The incidence of severe complications and mortality was found to be between 0.3 and 0.57% and 0.01, respectively (129–131). Furthermore, the interpretation of the biopsy requires a high level of expertise and training; hence experienced physicians need to perform it. Liver biopsies are also prone to sampling error with discordance of one stage or more of 41% in a study with paired biopsies (132). This is due to the fact that a biopsy sample is only 1:50.000 of the liver mass. Fibrosis is not spread uniformly throughout the liver, which leads to this sampling error (132). Another problem in the assessment of histological liver biopsy samples is inter- and intra-observer variability (128). Evaluation of fibrosis is mostly consistent among observers. The evaluation of inflammatory activity, on the other hand, was inconsistent at a high rate in a study performed by Younossi et al. (133). Moreover, NASH can mimic other liver diseases, therefore the possibility of another etiology needs to be kept in mind (117).

### Non-invasive Tests for the Detection of Liver Fibrosis

Non-invasive assessment of liver fibrosis can overcome some of the limitations of the biopsy and can be implemented and used for screening of NAFLD. There is currently an intensive search for biomarkers in NAFLD. Although stand-alone biomarkers are unlikely to provide the complex set of information that a liver biopsy offers, they can, if accurate and validated, provide an alternative to the biopsy to assess specific aspects of the disease. As outlined before, liver fibrosis is one of these crucial features, and non-invasive assessment of liver fibrosis has made significant advances in the last two decades. Currently, non-invasive assessment of liver fibrosis is composed of two different approaches: a biological approach based on the quantification of biomarkers (mostly in serum) and a physical approach based on the measurement of liver stiffness (117). A combination of the biological and physical approach results in a greater accuracy compared to the individual strategies to identify liver fibrosis, without the necessity of doing a liver biopsy (134, 135).

#### Methodological Aspects of Non-invasive Tests

As outlined previously, for a correct interpretation of the data, one should first of all look at the fibrosis scoring system that has been used in the design and validation of the non-invasive test. Hence, the non-invasive tests for NAFLD should be tested against the NASH CRN or SAF grading systems and not the MetaVir. Though, one should keep in mind the differences between the NASH CRN or SAF score. Moreover, as the non-invasive tests are validated against a liver biopsy, they cannot outperform the golden standard. Second, the values of non-invasive scores mostly show substantial overlap between histological fibrosis stages. Therefore, although often proposed for that purpose, non-invasive scores are not very accurate in predicting a precise corresponding histological fibrosis stage and hence cannot be used to diagnose the histological fibrosis stage of a given patient. According to the cut-off chosen, based on a given combination of specificity and sensitivity, non-invasive scores are useful to rule-in or rule-out significant or advanced fibrosis or cirrhosis, or conversely, the absence thereof, with NPV and PPV depending on the prevalence of the condition in the population that is studied. So, the result of a non-invasive test informs you about the likelihood of finding e.g., significant fibrosis, or the absence thereof, in a given patient, but does not tell you the patient has F2.

#### Liver Stiffness Measurement

##### Vibration Controlled Transient Elastography

The physical approach to assess fibrosis consists of measuring liver stiffness, which is a physical characteristic of the liver tissue, influenced by (but not equalling) the stage of liver fibrosis. Liver stiffness can be assessed by VCTE^TM^, as measured by the FibroScan® device, was shown to correlate with liver fibrosis in a cross-sectional analysis of patients with viral hepatitis and is now widely used as a technique to non-invasively assess liver fibrosis in various liver diseases and different circumstances, including not only screening and baseline assessment but also follow-up and assessment of treatment response (136, 137). VCTE^TM^ measures liver fibrosis via the velocity of a low-frequency (50 Hz) elastic shear wave (induced by a mechanical pulse) propagating through the liver (117). The probe uses pulse-echo ultrasound (US) to follow the propagation of the shear wave and measures its velocity. The velocity of the wave depends, amongst others, on the amount of liver fibrosis. It is a straightforward, non-invasive, and easy to use technique. The area covered by the VCTE^TM^ measurement has a volume that is 100 times bigger than an average liver biopsy sample (138). Choosing the cut-off value for the VCTE^TM^ has to be done with care and depends on the clinical situation. Low cut-off values for the VCTE^TM^, for example 7.9 kPa, have higher negative predictive values (NPV) than positive predictive values (PPV), meaning it can more precisely rule out more severe stages of fibrosis and rule in the absence of fibrosis. In contrast, higher cut-offs have an increase in the PPV and can, therefore, be more reliably used to rule-in more severe stages of fibrosis (139). A recent meta-analysis by Hsu et al. using the following thresholds 6.2, 7.6, 8.8, and 11.8 kPa, showed a pooled area under the receiving operating curve (AUROC) of 0.82, 0.87, 0.84, and 0.83 (with 95% CI) for diagnosing ≥F1, ≥F2, ≥F3, and F4, respectively (Table 2) (140).

**Table 2:** Overview of the accuracy indices of the different non-invasive diagnostic tools for NAFLD-related liver fibrosis.

**Non-invasive test**	**References**	**Meta-analysis**	**Fibrosis stage**	**Cut-off**	**AUROC (95% CI)**	**Sensitivity (%)**	**Specificity (%)**	**PPV (%)**	**NPV (%)**
VCTE^TM^	Hsu et al. (140)	Yes	≥F1	6.2 kPa	0.82 (0.76–0.88)	66	67.	81	48
			≥F2	7.6 kPa	0.87 (0.81–0.91)	76	80	72	83
			≥F3	8.8 kPa	0.84 (0.78–0.90)	77	78.	54	91
			≥F4	11.8 kPa	0.83 (0.74–0.94)	80	81.	34	97
MRE	Liang and Li (141)	Yes	≥F1	Optimal values could not be determined	0.89 (0.86–0.92)	77	90	N.A.	
			≥F2		0.93 (0.90–0.95)	87	86		
			≥F3		0.93 (0.90–0.95)	89	84		
			≥F4		0.95 (0.93–0.97)	94	75		
pSWE	Jiang et al. (142)	Yes	≥F2	Optimal values could not be determined	0.86	70	84	N.A.	
			≥F3		0.94	89	88		
			≥F4		0.95	89	91		
APRI	Peleg et al. (143)	No	≥F3	1	0.83	78	82	N.A.	
NFS	Xiao et al. (144)	Yes	≥F2	−1.1	0.72^*^ (0.65–0.79)	66^*^	83^*^	82^*^	74^*^
			≥F3	−1.455	0.78^*^ (0.75–0.81)	73^*^	74^*^	50^*^	92^*^
			≥F4	−0.014	0.83^*^ (0.76–0.89)	80	81	43	96
FIB-4	Xiao et al. (144)	Yes	≥F2	0.37–3.25	0.75^*^ (0.70–0.79)	64^*^	70^*^	73^*^	61
			≥F3	1.51–2.24	0.80^*^ (0.77–0.84)	77^*^	79^*^	66^*^	84^*^
			≥F4	1.92–2.48	0.85^*^ (0.81–0.89)	76^*^	82^*^	39^*^	96^*^
ELF	Vali et al. (145)	Yes	≥F2	7.7	0.81 (0.66–0.89)	93	34	N.A.	
FibroMeter^NAFLD^	Boursier et al. (146)	No	≥F2	N.A.	0.76	Not available			
			≥F3	0.311	0.76	80	62	65	83
			≥F4	N.A.	0.78	Not available			
FIBC3	Boyle et al. (147)	No	≥F3	>-0.4	0.89	83	80	74	88
NIS-4	Harrison et al. (148)	No	Exclude NAS≥4 and ≥F2	0.36	0.80 (0.77–0.84)	81	63	N.A.	78
			Include NAS≥4 and ≥F2	0.63		87	51	79	N.A.

* Mean values.

Table based on the most recent meta-analyses if available, or otherwise on the most robust studies.

*VCTE^TM^, vibration controlled transient elastography; MRE, magnetic resonance elastography; pSWE, point shear wave elastography; NFS, NAFLD fibrosis score; FIB-4, fibrosis-4 score; ELF, enhanced liver fibrosis score; FIBC3, fibrosis C3 panel; kPa, kilopascal; CI, confidence interval; PPV, positive predictive value; NPV, negative predictive value; N.A., not available*.

VCTE^TM^ has been found to be a cost-effective surveillance strategy to evaluate the presence of fibrosis (138). However, there are some limitations when using VCTE^TM^ measurements. Factors influencing the results of the FibroScan® measurements are ascites, elevated central venous pressure, and obesity. Gross ascites prevents an accurate measurement of liver stiffness by VCTE^TM^. Fluid and adipose tissue attenuate the elastic wave (149–151). To overcome the latter problem, the extra-large (XL) probe was developed. It is able to assess the degree of fibrosis more accurately, though it may not be superior to the standard medium probe in obese patients (152, 153). The XL probe has a more sensitive US transducer, larger vibration amplitude, deeper focal length, and deeper signal penetration (tissue depth >35–75 mm) (154).

Liver inflammation may also reduce the accuracy of the test, as it can increase the VCTE^TM^ value by 1.3 to 3 times. This is illustrated by the rapid decline of liver stiffness after successful eradication of viral hepatitis C in a time frame that is too short to allow for substantial fibrosis regression (155). The pattern of fibrosis also differs between diseases, and the staging systems differ accordingly, as outlined previously. Accuracy and cut-offs need hence to be defined in a disease-specific way. Operator experience, sex, and metabolic syndrome can influence the FibroScan® measurements too (156, 157). A study performed by Vuppalanchi et al. demonstrated a failure rate of 5.5% because of excess skin to liver capsule distance, machine error, and invalid readings. Another study performed by the same research group indicated that fasting of at least 3 h in necessary (158). Without fasting, a significant increase (26 ± 25%, *p* = 0.02) in VCTE^TM^ was seen (159). Nonetheless, with sufficient operator experience, the failure rate and unreliability can be minimized (117, 160).

An extra feature recently added to the FibroScan® device is the possibility to measure the amount of liver fat. Since this an important characteristic of NAFLD, assessment of steatosis is therefore crucial. The fat content can be measured via the Controlled Attenuation Parameter (CAP^TM^). The CAP^TM^ can be determined by the ultrasonic attenuation on the FibroScan® device at a frequency of 3.5 MHz on a go-and-return path (161, 162).

##### Other Ultrasound-Based Elastography Methods

There are several other ultrasound (US)-based methods available to determine liver elasticity (163). US elastography makes use of two different techniques, namely strain imaging or shear wave imaging. Strain imaging is used with strain elastography (SE) and acoustic radiation force impulse (ARFI) (164). Shear-wave imaging is the same technique as in the FibroScan® device (165). They have been less extensively studied in the context of NAFLD, but data on their accuracy are increasingly reported along with their use in clinical practice (166).

Point shear wave elastography (pSWE) is an ARFI-based technique that uses a short-duration, high-intensity acoustic pulse to displace tissue perpendicular to the longitudinal waves of the tissue surface (167). Next, the transducer detects the tissue displacement within a focal point along the radiation force resulting in the measurement of tissue stiffness. The big advantage of pSWE is that additional equipment is not necessary. pSWE can be incorporated in an US machine with brightness-mode. Next, direct anatomical visualization is possible, avoiding the areas with large blood vessels or parts of the biliary system (168). This implies, however, that a radiologist or sonographer is usually needed to perform the pSWE as a specific anatomical and technical expertise is necessary to interpret the visual images (169). Furthermore, in contrast to VCTE^TM^, pSWE has a lower failure rate of 1–2% due to the fact that it is not limited by the presence of ascites (142, 170). A meta-analysis that compared VCTE^TM^ and pSWE showed that both provide excellent diagnostic accuracies for the diagnosis of advanced fibrosis and cirrhosis (Table 2) (142). However, a recent study conducted by Leong *et al*. comparing VCTE^TM^ with pSWE for diagnosis of fibrosis stage in a biopsy-proven cohort found that VCTE^TM^ outperformed pSWE. (171). Especially for the diagnosis of ≥F2 and ≥F3, the AUROC for VCTE^TM^, respectively, 0.83 and 0.83, was higher than that of pSWE (0.72 and 0.69) (171).

##### Magnetic Resonance Elastography

Magnetic resonance elastography (MRE) is a magnetic resonance imaging-based method for quantitatively imaging tissue stiffness. These measurements can be taken rapidly during breath-hold acquisition mode. Even in the early stages, MRE can be used to detect NAFLD. The diagnostic accuracy of MRE for liver fibrosis and steatosis is higher than VCTE^TM^ and CAP^TM^ (172). The pooled summary receiver operating characteristics (SROC) curve of MRE in 12 studies, including 910 patients with biopsy-proven NAFLD, was 0.89 for ≥F1, 0.93 for ≥F2, 0.93 for ≥F3, and 0.95 for F4, respectively (Table 2) (141). Nonetheless, due to high-performance costs, MRE is usually not performed routinely to screen patients for NAFLD (172).

#### Non-invasive Score Calculations to Detect Liver Fibrosis

The biological approach via non-invasive score calculation is composed of routinely measured clinical and laboratory variables that can aid in predicting liver fibrosis. Different scores have been proposed to calculate the risk of fibrosis. The aspartate aminotransferase (AST)-to-platelet ratio index (APRI), developed initially for hepatitis C infection, has been suggested for predicting significant fibrosis in NASH (117, 143). The NAFLD Fibrosis Score (NFS) has been demonstrated to be useful as a prognostic marker for fibrosis. Advanced fibrosis can be excluded with an NPV of 93% when using a low cut-off value and having a high PPV 90% (117). A meta-analysis conducted by Xiao et al. found an AUROC value of 0.78 for the exclusion of advanced fibrosis (Table 2) (144). The Fibrosis-4 (FIB-4) score was also designed as a parameter of fibrosis detection in patients with hepatitis C infection. This index is not influenced by BMI and is composed of routinely available laboratory data (AST, alanine aminotransferase (ALT) and platelets) (117). The FIB-4 had an AUROC of 0.80 for diagnosing advanced fibrosis, a sensitivity of 77%, a specificity of 79%, a PPV of 66%, and an NPV of 84% (Table 2) (144). The Enhanced Liver Fibrosis (ELF) score developed for the detection of liver fibrosis has good accuracy for the non-invasive diagnosis of advanced fibrosis in NAFLD (145). The ELF test is a panel consisting of the following markers: PIIINP, HA, and TIMP1 (173, 174). One side note, although it still has the same name, the test components and formula have been altered throughout the years. Nevertheless, not all studies reflect the accuracy of the current ELF test (145). A recent meta-analysis by Vali et al. in biopsy-proven NAFLD patients examined the accuracy of the ELF test for the diagnosis of advanced and significant liver fibrosis and NASH (145). At the recommended cut-off of 7.7, a high sensitivity (93%) was found, though specificity was limited. When the high cut-off was used (9.8), a higher specificity of 86% was reached. The cut-off value should thus be decided based on the purpose of the test in a specific clinical situation (the so-called context of use) (145).

FibroMeters, a family of blood tests specifically designed for each cause of chronic liver disease, were commercialized by Echosens (175). Although a FibroMeter^NAFLD^ is available, the FibroMeter^V2G^, developed for hepatitis C is more accurate in NAFLD with an AUROC of respectively 0.76 and 0.8 for the detection of F≥3 (Table 2) (146). This is probably due to the fact that the FibroMeter^NAFLD^ only contains AST, ALT, platelets, glucose, and ferritin, whereas the FibroMeter^V2G^ uses AST, urea, platelets, prothrombin time, HA, and A2M. These last two are direct markers of liver fibrosis, while the others are indirect markers (146). In a biopsy-proven NAFLD cohort, the FibroMeter^VCTE^ was tested for accuracy. FibroMeter^VCTE^ combines the results of the VCTE^TM^ and FibroMeter^V2G^ markers in one test. In this cohort, the FibroMeter^VCTE^ was significantly more accurate than the FibroMeter or VCTE^TM^ alone (AUROC: 0.87 ± 0.012, *p* ≤ 0.005) (135).

Other parameters or scores that have been proposed for the detection of NAFLD related liver fibrosis are PRO-C3 and NIS4. Measurement of type III collagen neo-epitopes (PRO-C3) as a single diagnostic marker or as part of a panel has shown to have reasonable accuracy in assessing NAFLD disease stage and activity (147). When used as a single marker, PRO-C3 performed equally to simple panels like the FIB-4 (176). This might be due to the fact that PRO-C3 is more a product of active fibrogenesis instead of static collagen accumulation. PRO-C3 might therefore be helpful to detect patients with active liver fibrogenesis (177, 178). When on the other hand, used in the FIBC3 panel in combination with age, BMI, T2DM, platelets, it was able to distinguish advanced fibrosis (≥F3) with an AUROC of 0.89, a specificity of 80%, sensitivity of 83%, PPV of 74% and an NPV of 88%, respectively, for a cut-off value of >-0.4 (147). In three independent cohorts with suspected NASH, the non-invasive blood-based diagnostic test NIS-4 was developed and validated to detect patients with NAS≥4 and ≥F2 (148). The NIS4 panel comprised of the following NASH-associated biomarkers: miR-34a-5p, A2M, YKL-40, and glycated hemoglobin (HbA1c) (179, 180). The exact functions of miR-34a-5p and YKL-40 in the development of fibrosis are not yet fully understood, though their levels are elevated in patients with liver fibrosis (181–183). In the pooled validation cohort, NAS≥4 ≥F2 patients were excluded with a cut-off of 0.36 and this with a sensitivity and specificity of 81.5 and 63%, respectively. To include NAS≥4 and ≥F2 patients, a NIS4 value of 0.63 was used. This resulted in a sensitivity of 87.1% and a specificity of 50.7%. Furthermore, the NIS4 algorithm experiences no influence of age, sex, BMI, or liver enzyme concentrations (148).

Nonetheless, scoring systems have their limits. There is no single threshold for non-invasive tests that has the perfect balance between sensitivity and specificity. Up to now, the scores are more used as a first-line risk determination, without the necessity of doing a liver biopsy. For example, the NFS works best in distinguishing advanced vs. non-advanced or any with no fibrosis (172). In 25 to 30% of the patients, however, the NFS score is intermediate (117). A recent study indicated that the NFS and FIB-4 scores were better compared to the other scores (BARD, APRI, and AST/ALT ratio) to determine fibrosis, and as good as MRE in predicting the presence of advanced fibrosis in patients with biopsy-proven NAFLD (172).

#### Future Biomarkers for Liver Fibrosis

Up to now, no accurate serum biomarkers are available to detect a precise stage of fibrosis. Fortunately, a lot of research is carried out on this topic. For example, Mac 2-binding protein glycan isomer (M2BPGi) is secreted by HSCs to act as a messenger for Kupffer cells during fibrosis progression (184). M2BPGi can therefore act as biomarker for detection of liver fibrosis (185). A study by Nah and colleagues indicated that M2BPGi can exclude advanced fibrosis with a sensitivity of 80% and specificity of 77.9% and a NPV of 98.9% with an AUROC of 0.85 when compared to MRE (186). Serum autotaxin (ATX) may also be a potential serum biomarker for liver fibrosis with NAFLD ADDIN EN.CITE (187, 188). ATX is responsible for the transformation of lysophosphatidylcholine to lysophosphatidate (189). The latter is involved in the process of cell migration, neurogenesis, angiogenesis, smooth muscle contraction, platelet aggregation, and wound healing (190, 191). Sinusoidal endothelial liver cells process ATX, therefore, it is thought that in the case of chronic liver injury, ATX metabolism is impaired. First results within a cohort of NAFLD patients with fibrosis show that ATX can select patients who require further evaluation. The diagnostic accuracy was, however, lower than that of MRE (187). More recently a study was published by Kimura *et al*. on the possible biomarker thrombospondin 2 (TSP2) for the detection of liver fibrosis with NAFLD. TSP2 is involved in multiple processes such as collagen/fibrin formation. TSP2 had an AUROC of 0.82 for prediction of ≥F3 (192). Lastly, the serum marker type IV collage 7s can be used to diagnose significant fibrosis with an AUROC of 0.832, a sensitivity of 91.4%, and a NPV of 87.9% with a cut-off value of ≥5.2 ng/mL (193).

A recent study by Caussy et al. demonstrated that a combination of 10 metabolites consisting of eight lipids (5α-androstan-3β monosulfate, pregnanediol-3-glucuronide, androsterone sulfate, epiandrosterone sulfate, palmitoleate, dehydroisoandrosterone sulfate, 5α-androstan-3β disulfate, and glycocholate), one amino acid (taurine), and one carbohydrate (fructose) could detect advanced fibrosis. With an AUROC of the metabolite combination of 0.94 and a sensitivity and specificity of, respectively, 90 and 79%, the metabolites performed better than the FIB-4 (0.78) and NFS (0.84) for the detection of advanced liver fibrosis (194). However, they used mass spectrometry to analyse the metabolites, which is not easily accessible and expensive and therefore not (yet) applicable in a routine clinical situation (195). However, this type of biomarkers, requiring more sophisticated techniques and all kinds of omics approaches, might show the way ahead to increase the accuracy over the currently available tools. To support biomarker development for the detection of liver fibrosis, two large projects, one in the United States of Amerika called Non-Invasive Biomarkers of Metabolic Liver Disease (NIMBLE), and one in Europe called the Liver Investigation: Testing Marker Utility in Steatohepatitis (LITMUS), have been set-up (196).

#### The Use of Non-invasive Biomarkers in a Clinical Situation and in Trials

A non-invasive test alone has a certain accuracy depending on the context of use (147). Notwithstanding, when used in a sequential way or at the same time, the accuracy of the non-invasive tests increases significantly. The most appropriate combination is probably one with a biological test in combination with liver stiffness measurements. In a recent study by Boursier et al. different stepwise combinations were tested. The sequential combinations of the FIB-4 followed by FibroMeter^VCTE^ and the VCTE^TM^ followed by the FibroMeter^VCTE^ provided a diagnostic accuracy of 90%. A liver biopsy to confirm the results was only needed in 20% of the cases (135). Another study conducted by Srivastava et al. in a primary care cohort tested a 2-step algorithm that combined the FIB-4, followed by, if needed, the ELF test (197). Use the 2-step algorithm improved detection of advanced fibrosis and cirrhosis by 4.9-fold (197). Davyduke et al. piloted a FIB-4 first strategy, followed by a VCTE^TM^ when classified as high risk (198). When using this strategy, only 15% of the patients needed to be referred for further assessment (198). With a probabilistic decision model of a cohort of 1000 NAFLD patients, different sequential combinations of the non-invasive tests were simulated, and costs were compared. The price per case of advanced fibrosis was significantly lower when using a sequential combination (£8,932 for FIB-4/ELF, £9,083 for FIB-4/VCTE^TM^) compared to the standard of care (£25,543) (199). Proving that the sequential combinations of non-invasive tests are cost-effective, reduce unnecessary referral and detect advanced fibrosis without the necessity of doing a liver biopsy, which can be useful for inclusion in clinical trials (199–201).

Currently, efficacy assessment in clinical trials requires histology hence biopsy in phase II trials that need to provide data to go into phase III, and for the interim analysis for conditional approval in phase III, with regression of fibrosis of 1 stage without worsening of fibrosis as one of the endpoints for regulatory approval in non-cirrhotic NASH (119, 120). Efficacy assessment in earlier phase II trials, on the other hand, can be based on non-invasive biomarkers, and trial sponsors are encouraged to collect data on biomarker response in late phase II and in phase III trials to inform future trial design. Several non-invasive markers of fibrosis have been used in several trials and mostly serve to support the data on histology. For example, in the pirfenidone (PFD) study, they evaluated the antifibrotic effects with, similarly, for the cenicriviroc (CVC) study where they used the NFS, FIB-4, APRI, and ELF scores (196, 202).

Much more data and analyses are needed to couple responses in histology to responses in biomarkers and to define criteria of response in terms of biomarkers, i.e., what magnitude of change, absolute and or relative, is clinically meaningful and correlates with a histological response or with another endpoint concerned clinically relevant and resulting in clinically significant benefit.

## Anti-Fibrotic Drugs

NAFLD management is centered on lifestyle modifications, weight loss, and habitual physical activity. Weight loss promotes fat reduction and NAFLD remission. A bodyweight reduction of 3 to 5% improves steatosis, and a decrease of 10% improves necroinflammation and fibrosis (1). However, dietary and lifestyle changes are hard to maintain. As a result, there is a need for appropriate drugs to treat NAFLD. The target of treatment is still a matter of debate. Fibrosis regression (mostly defined by at least one stage improvement according to NASH CRN), as a highly potential surrogate marker for clinical benefit, is one of the endpoints approved by the regulatory authorities for phase III trials in NASH (119, 120). Unfortunately, there are currently no drugs approved specifically for the treatment of liver fibrosis by the United States of America Food and Drug Administration (FDA) or European Medicines Agency (EMA) (203), despite the increased insight into the molecular and cellular mechanisms of liver fibrosis.

### Current Options for NAFLD Treatment

Besides vitamin E, the only drugs that can be recommended for the treatment of NAFLD, are drugs already used for the treatment of T2DM treatment and/or obesity. Most of these drugs have a direct effect on NASH, and via this, an indirect effect on fibrosis regression (although direct antifibrotic effects might even so be present) since NASH and fibrosis are strongly intertwined.

Drugs used to treat T2DM with effects on NAFLD histology are thiazolidinediones (TZDs), glucagon-like peptide-1 receptor agonist (GLP-1 RA) and sodium-glucose cotransporter- (SGLT)-2 inhibitors (117, 203–205). TZDs like pioglitazone act on peroxisome proliferator-activated receptors (PPARs), mainly on the PPARγ isoform. If PPARγ binds to the retinoid X receptor, it has powerful insulin-sensitizing properties in adipose tissue. Although PPARγ is poorly expressed in the hepatocytes, it still exerts anti-steatogenic effects (206). In rat livers, depletion of PPARγ led to a decrease in fibrogenesis (207). However, the effect was not so strong in human trials with TZDs. TZDs cause a reduction in liver fat, despite some overall weight gain, which is reflective of an improvement in adipose tissue function and goes along with a redistribution of fat from visceral to subcutaneous fat storage (208). PPARγ is also implicated in the activation state of HSC, so TZDs can also have direct effects on fibrogenesis. Based on the histological improvements seen with pioglitazone, several guidelines recommend the use of TZDs in patients with liver biopsy-proven NASH and T2DM (209). In the PIVENS trial, after 96 weeks of pioglitazone treatment, no improvement in fibrosis stage was seen when compared to placebo (210).

GLP-1 RAs improve glycaemic control via a decreased glucagon secretion, slowed gastric emptying, glucose-dependent insulin secretion, enhanced satiety, reduction in body weight, and BMI (204, 211–213). They also lead to the improvement of the hepatic markers ALT, AST, and gamma-glutamyl transferase (GGT). Presently, GLP-1 RA use is only recommended in case of a high BMI (>27 kg/m^2^) and comorbidities like diabetes and arterial hypertension (AHT). However, NASH should be added to the list of comorbidities associated with obesity (117). In the LEAN trial, the efficacy of 48 weeks of liraglutide, a GLP-1 RA, was investigated with NASH regression without worsening of fibrosis as the primary endpoint. Despite the significant difference in the resolution of NASH between liraglutide and placebo, no significant difference in fibrosis score was detected (214). Another GLP-1 RA, semaglutide, was tested in patients at risk for NAFLD development for 104 weeks. Semaglutide significantly reduced ALT after 28 to 20 weeks of treatment (215). In a recent study by Legry et al. the effect of semaglutide in mice with induced NASH was researched. This showed a reduction in NAS though it did not reduce fibrosis (216). These results were confirmed in human trials, semaglutide was significant on the resolution of NASH, though not on the fibrosis endpoint after 72 weeks of treatment (217). Despite having an efficacy on NASH resolution, the TZD pioglitazone and GLP-1 RA semaglutide were not able to reach significance on the ≥1 point fibrosis reduction endpoint. Notwithstanding, a decrease in the mean fibrosis score has been reported (218, 219).

SGLT-2 is a class of oral antidiabetics that reduces hyperglycaemia by promoting urinary excretion of glucose without affecting insulin secretion (220). In rodent models of T2DM, the SGLT-2 inhibitor ipragliflozin prevented the development of NASH (221). Not only in rodent models SGLT-2 inhibitors proved their effect of NAFLD development, in patients with T2DM who received dapagliflozin or empagliflozin a decrease in hepatic steatosis was seen. However, in these studies, no effects on liver fibrosis were detected (205, 222). A recent meta-analysis by Mantovani et al. also confirmed the significant effect on hepatic steatosis, though up to now, no results on the histological response of SGLT-2 inhibitors are available, at least not from randomized placebo-controlled trials (223, 224).

### Therapy in Development

As mentioned above, there are currently no approved drugs for NAFDL treatment, yet potent drugs are coming. The first drugs are already in phase III trials and are expected to be on the market by the end of 2020 (117). As mentioned, fibrosis regression of at least one point without worsening of NASH, as a likely reasonable surrogate for a clinically meaningful benefit, is one of the two regulatory endpoints for conditional approval in non-cirrhotic NASH. Obeticholic acid (OCA) is so far the only drug that demonstrated efficacy on this endpoint in phase III. OCA is a first-in-class selective farnesoid X receptor (FXR) agonist with anti-cholestatic and hepato-protective properties (225). The FXR is a bile acid nuclear receptor that plays a role in lipoprotein and glucose metabolism, hepatic regeneration, and regulation of hepatic inflammation. Activation of the FXR receptor in mice has been shown to inhibit NLRP3 inflammasome activation in hepatocytes, thereby preventing disease progression (226). OCA was FDA approved in 2016 for the treatment of primary biliary cholangitis, though it is currently in a phase III trial to test the effects and safety in NAFLD (Regenerate Study) (209). In a first interim analysis of the Regenerate study, OCA thus demonstrated statistically significant fibrosis regression of at least one point with an effect size of 11% in phase III after 72 weeks on 25 mg. OCA did, however, not meet the endpoint of NASH resolution (227).

Other drugs that are currently being tested for the treatment of fibrosis are lanifibranor, PFD, and CVC. Lanifibranor (IVA337) is a drug that activates each of the three PPAR isoforms (228). These isoforms play an essential role in the regulation of cellular differentiation, development, and tumorigenesis throughout the body. The drug has both anti-fibrotic and anti-inflammatory effects and is also beneficial for metabolic changes. Currently, lanifibranor is going into phase III, based on the significant results the drug demonstrated on both resolution of NASH and improvement of fibrosis and the combination of both (229–231). PFD is an oral antifibrotic drug approved for the treatment of idiopathic pulmonary fibrosis. In a recent study by Poo et al. the effect of prolonged-release formulation (PR-PFD) plus standard of care was tested in patients with advanced liver fibrosis (PROMETEO Study). In 35% of the patients, a significant reduction of fibrosis was seen, leading to the conclusion that PR-PFD is efficacious and safe in patients with advanced liver disease. Moreover, it showed promising antifibrotic effects (196). CVC, a drug that targets macrophages in the liver by inhibiting the C-C chemokine receptors CCR2 and CCR5 (232), met this endpoint as a key secondary endpoint in phase II after 1 year of treatment with an effect size of 9.6% (*p* = 0.023) but was not significant at 2 years and showed no efficacy on NASH resolution (202, 233). A phase III trial (AURORA) is still ongoing evaluating the effect of CVC in NASH patients with F2 or F3 (234).

Different molecules failed, despite pre-clinical studies being positive. For instance, selonsertib is an apoptosis signal-regulating kinase 1 inhibitor that has been demonstrated in patients with moderate-to-severe NASH to reduce fibrosis, steatosis, and progression to cirrhosis (235). However, in several phase III clinical trials, selonsertib did not achieve the endpoints for the reduction of fibrosis (236). This also applies to simtuzumab, a monoclonal antibody against lysyl oxidase-like 2 that is involved in fibrogenesis. In the phase IIb trials, simtuzumab was unable to reduce fibrosis (237). Likewise, for the galactin-3 inhibitor GR-MD-02, phase II trials failed to show efficacy in NASH and reduction of liver fibrosis (238, 239). Correspondingly also elafibranor, a dual PPAR agonist that showed promising results in the reduction of NASH and liver fibrosis in a phase II trial, failed to replicate these results in a phase III trial. Therefore, the trial has been ended (240). Recent suggestions made by Ratziu et al. stated that these failed trials were caused by the rush to move compounds into clinical development without thoroughly being investigated in the pre-clinical trials (241). More attention should be paid to optimize treatment dose and regimen correctly, but most importantly, the results of small studies should be interpreted with care (241).

### Future Therapeutic Targets

Although recent clinical trials have been promising in treating NAFLD-related liver fibrosis, the overall efficacy of these drugs has been modest. Only a minority of patients achieved treatment response (242). Possible new targets can be found in the gut microbiome. As stated, it plays an important role in fibrogenesis. Therefore, targeting the GLA by modulating the gut-microbiome can be a promising therapeutic approach in NAFLD (243). In mice, whole-body deletion of nucleotide-binding oligomerization domain-containing (NOD)2 caused an increase in liver steatosis and fibrosis. NOD2 can therefore potentially engage the GLA to protect against steatosis, fibrosis, and gut dysbiosis (244). Another possible treatment option is the blocking of PDGF. Blocking of PDGF signaling ameliorates experimental liver fibrogenesis. PDGF signaling can be blocked by regulation of the isoforms, regulation of the receptor binding, and finally, by inhibiting the signaling pathways (245). PDGFR kinase activity blocking is one of the most efficient ways to block the signaling pathway of PDGF. Several kinase inhibitors have been developed, though they are not entirely specific. Imatinib mesylate (Gleevec®) effectively inhibits PDGFR signaling in CCl_4_-treated mice leading to improved liver regeneration *in vivo* and induced apoptosis of HSCs both *in vivo* and *in vitro* (246). In a pig serum-induced rat model of liver fibrosis, characterized by a slow progression of fibrosis, like in a human situation, imatinib had an effect in the early stages of liver fibrosis (247). Finally, Wnt3a, a canonical Wnt ligand, could be used as a future therapeutic target. In a study conducted by Wang et al. where LRP6 mutant mice were treated with Wnt3a, liver inflammation was reduced, indicating the ant-inflammatory role of Wnt3a (97).

## Conclusion

Progressive liver fibrosis in NAFLD can lead to cirrhosis and liver-related morbidity and mortality and is also the strongest predictor of overall mortality. Halting fibrosis progression and regression of existing fibrosis are hence essential goals for treatment. Liver biopsy is still the gold standard for the staging of liver fibrosis. Accurate non-invasive diagnosis of liver fibrosis that can replace liver biopsy in most of the circumstances is obviously needed, both for initial diagnosis and monitoring of evolution over time and response to treatment. Non-invasive biological markers and liver stiffness measurement, alone or in combination, are extensively studied and further developed and validated. Currently, anti-fibrotic drugs are in development, and some have promising results that can lead to the prevention of liver fibrosis and delaying or even halting the development of cirrhosis. Moreover, based on the available data and international guidelines, a multi-disciplinary approach to treat and guide NAFLD patients is recommended due to the association with other metabolic features. These developments will potentially have an extensive impact on global health and on the healthcare costs that accompanies the rising incidence of NAFLD-related fibrosis. Furthermore, it has the possibility to lower the increasing incidence of HCC. Nonetheless, a better understanding of the complex pathology of NAFLD-related fibrogenesis is necessary to identify new targets for treatment and to find markers that will lead to new diagnostic methods that can accurately detect disease severity and fibrosis stages and its evolution over time.

## Author Contributions

LH collected the data and drafted the first version of the paper. All authors listed have made a substantial, direct and intellectual contribution to the review, and approved it for publication.

## Conflict of Interest

DB has received travel grants from AbbVie and Gilead Sciences and research grants from Gilead Sciences. GR has received research grants from AbbVie, Janssen Pharmaceuticals, MSD, and has acted as a consultant/advisor for AbbVie, BMS, Gilead Sciences and MSD. The remaining authors declare that the research was conducted in the absence of any commercial or financial relationships that could be construed as a potential conflict of interest.
